# On the influence of the culture conditions in bacterial antifouling bioassays and biofilm properties: *Shewanella algae*, a case study

**DOI:** 10.1186/1471-2180-14-102

**Published:** 2014-04-23

**Authors:** Alberto J Martín-Rodríguez, Alejandro González-Orive, Alberto Hernández-Creus, Araceli Morales, Roberto Dorta-Guerra, Manuel Norte, Víctor S Martín, José J Fernández

**Affiliations:** 1 Institute for Bio-Organic Chemistry “Antonio González”, Center for Biomedical Research of the Canary Islands (CIBICAN), University of La Laguna, Avenida Astrofísico Francisco Sánchez 2, La Laguna, Tenerife 38206, Spain; 2 Department of Physical Chemistry, University of La Laguna, Avenida Astrofísico Francisco Sánchez 1, La Laguna, Tenerife 38206, Spain; 3 Department of Physiology, Institute of Biomedical Technologies, CIBICAN, University of La Laguna, Campus de Ofra, s/n, La Laguna, Tenerife 38071, Spain; 4 Department of Statistics, Operations Research and Computation, University of La Laguna, Avenida Astrofísico Francisco Sánchez 2, La Laguna, Tenerife 38206, Spain; 5 Oceanic Platform of the Canary Islands, Carretera de Taliarte s/n, Telde, Gran Canaria 35214, Spain; 6 Institute of Materials and Nanotechnology, University of La Laguna, Avenida Astrofísico Francisco Sánchez s/n, La Laguna, Tenerife 38206, Spain

**Keywords:** Biofilm, Biofouling, Antifouling, *Shewanella algae*, CLSM analysis, Atomic Force Microscopy, Young modulus, Adhesion forces

## Abstract

**Background:**

A variety of conditions (culture media, inocula, incubation temperatures) are employed in antifouling tests with marine bacteria. *Shewanella algae* was selected as model organism to evaluate the effect of these parameters on: bacterial growth, biofilm formation, the activity of model antifoulants, and the development and nanomechanical properties of the biofilms.

The main objectives were:

1) To highlight and quantify the effect of these conditions on relevant parameters for antifouling studies: biofilm morphology, thickness, roughness, surface coverage, elasticity and adhesion forces.

2) To establish and characterise in detail a biofilm model with a relevant marine strain.

**Results:**

Both the medium and the temperature significantly influenced the total cell densities and biofilm biomasses in 24-hour cultures. Likewise, the IC_50_ of three antifouling standards (TBTO, tralopyril and zinc pyrithione) was significantly affected by the medium and the initial cell density. Four media (Marine Broth, MB; 2% NaCl Mueller-Hinton Broth, MH2; Luria Marine Broth, LMB; and Supplemented Artificial Seawater, SASW) were selected to explore their effect on the morphological and nanomechanical properties of 24-h biofilms. Two biofilm growth patterns were observed: a clear trend to vertical development, with varying thickness and surface coverage in MB, LMB and SASW, and a horizontal, relatively thin film in MH2. The Atomic Force Microscopy analysis showed the lowest Young modulii for MB (0.16 ± 0.10 MPa), followed by SASW (0.19 ± 0.09 MPa), LMB (0.22 ± 0.13 MPa) and MH2 (0.34 ± 0.16 MPa). Adhesion forces followed an inverted trend, being higher in MB (1.33 ± 0.38 nN) and lower in MH2 (0.73 ± 0.29 nN).

**Conclusions:**

All the parameters significantly affected the ability of *S. algae* to grow and form biofilms, as well as the activity of antifouling molecules. A detailed study has been carried out in order to establish a biofilm model for further assays. The morphology and nanomechanics of *S. algae* biofilms were markedly influenced by the nutritional environments in which they were developed. As strategies for biofilm formation inhibition and biofilm detachment are of particular interest in antifouling research, the present findings also highlight the need for a careful selection of the assay conditions.

## Background

Biofouling is a colonisation process that begins from the very same moment a material surface is immersed in seawater and leads to the development of complex biological communities. This undesirable accumulation of biological material causes severe economic losses to human activities in the sea, from deterioration of materials, structures, and devices, to increases in fuel consumption and loss of maneuverability in ships [1,2]. In a simplified model, there are four main stages in the biofouling process: i) adsorption of organic matter onto the material surface, creating a conditioning film; ii) arrival of the so-called primary colonizers (bacteria and diatoms, mainly) that form complex, multispecies biofilms; iii) settlement of spores of macroscopic algae and other secondary colonisers; and iv) settlement of invertebrate larvae [3]. Even though it is not necessarily a sequential process, it is generally accepted that the formation of an organic layer and a biofilm is the first step to biofouling [4].

Since the ban on the use of organotin compounds, particularly bis-(tris-*n*-butyltin) oxide (TBTO), established by the International Maritime Organization (IMO) that finally entered into force in September 2008, there is a clear need for alternative antifouling compounds. We have recently started a screening program for the search of novel antifouling molecules. In doing so, one of the most striking issues is the great diversity of conditions currently employed in lab-scale assays (i.e., culture media, inocula, incubation times and temperatures), not only when dealing with biofilms, in whose case the optimal conditions should be individually defined for each strain, but even with planktonic cultures [5-11]. It seems evident that this heterogeneity may lead to important differences in the results obtained from *in vitro* tests. In addition, there is a lack of studies focusing on the effect that these diverse conditions have on the properties of marine biofilms.

**Figure 1:**
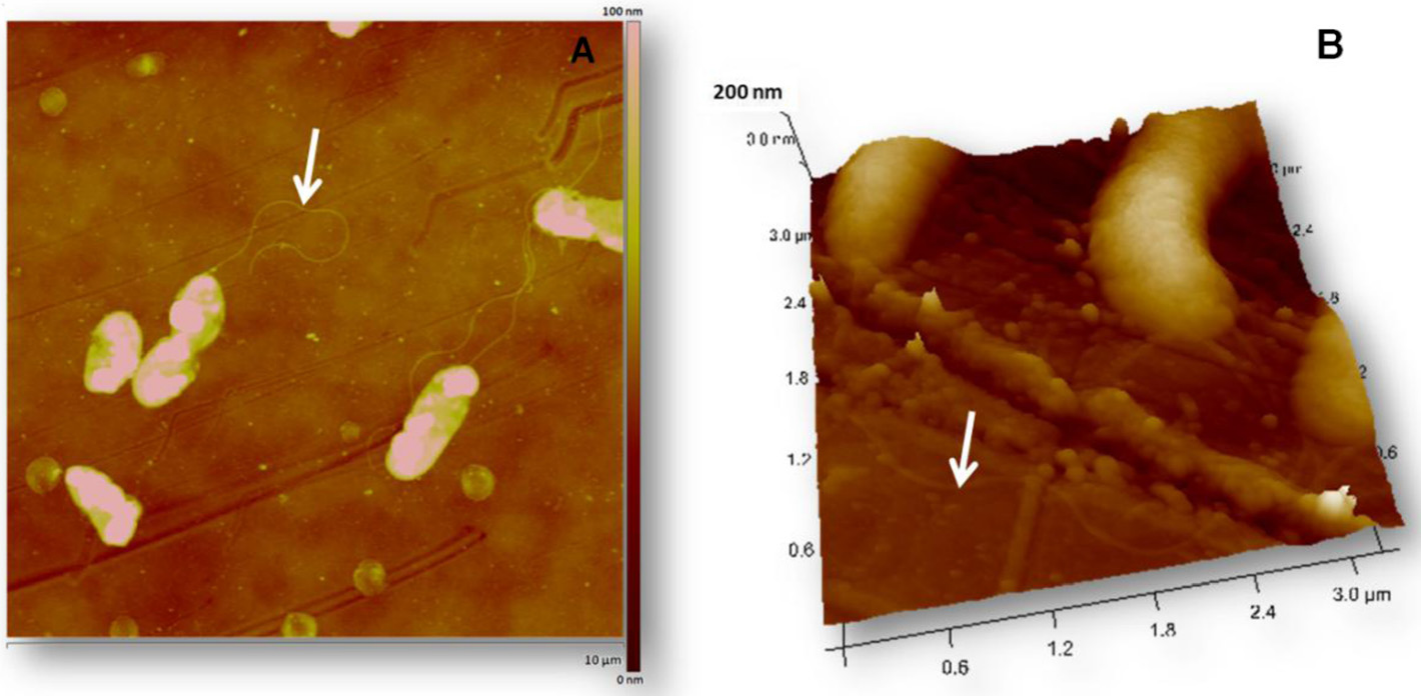
**Tapping mode images in air of *Shewanella algae *adsorbed on treated polystyrene. (A)** 10 × 10 μm^2^ bidimensional image showing bacterial dimensions and their characteristic flagella; **(B)** 3.2 × 3.2 μm^2^ three-dimensional image with bacterial surface roughness and flagella in detail. White arrows indicate the position of flagella.

Even though single-strain laboratory tests do not mimic the real environmental conditions, *in vitro* models are a useful tool for screening and comparing new products, treatments and materials. To this end, *S. algae* was chosen as model organism. *Shewanella* spp. are gram-negative, facultative anaerobe rod-shaped uniflagellar bacteria worldwide distributed in marine and even freshwater habitats (Figure 1) [12,13]. They play an important role in the biogeochemical cycles of C, N and S [13] due to their unparalleled ability to use around twenty different compounds as final electron acceptors in respiration, which, in turn, provides bacteria the ability to survive in a wide array of environments [14]. For this versatility, shewanellae have been focus of much attention in the bioremediation of halogenated organic compounds, nitramines, heavy metals and nuclear wastes [14]. Their biofilms are also receiving increasing interest in microbial fuel cell applications due to their excellent extracellular electron transfer capability, that includes direct (e.g. nanowires) and indirect mechanisms (electron shuttles such as flavins) [15]. *Shewanella* spp. biofilms have been found to modulate the settlement (with inductive or inhibitory effects) of a variety of macroscopic algae and invertebrates such as *Ulva* spores [16-18], cypris [19], mussel larvae [20], or sea urchin larvae [21]. *Shewanella* spp. produce omega-3 fatty acids and other hydrocarbons, probably to increase the fluidity of the cell membrane in cold waters –most *Shewanella* strains are psychrotolerant- or as a result of a mutualist relationship between fish and bacteria living in their intestines [14,22]. Indeed, they are being increasingly used as probiotics in aquaculture [23,24] and, more recently, as a source of hydrocarbon fuels [22]. Among all the members of the shewanellae family, only *S. putrefaciens* and *S. algae* are widely recognized to be pathogenic to human and animals, being involved in soft-tissue infections, ear infections, necrotising fasciitis, abscesses, bacteremia, and many other affections [12,25-29]. However, there is increasing evidence that point that other *Shewanella* species are also causative agents of human infections [30,31]. For all these reasons, *S. algae* biofilms are of great interest in bacterial fouling studies as well as in many other fields.

In anti-biofilm assays, the nutritional requirements that promote bacterial biofilm formation may not be the same as those employed in antimicrobial susceptibility testing, thus leading to the use of a different culture medium and frequently higher inocula [32]. In order to explore the effect of the culture conditions on the growth and biofilm formation of *S. algae*, nine media and two incubation temperatures were initially screened. Subsequently, the antibacterial activity of known antifouling biocides was determined using different media and inocula. Finally, in order to assess exhaustively the morphological and physical properties of *S. algae* biofilms developed in different media, a detailed examination was conducted by Confocal Laser Scanning Microscopy (CLSM) and Atomic Force Microscopy (AFM). Over the last few years, AFM has turned into a powerful technique not only for studying the morphology of soft materials such as polymers and biomaterials but also for obtaining information about different properties (mechanical, electrical, magnetic, etc.) of the samples. In particular, recent advances in force distance-based AFM (FD-AFM) have allowed researchers to register three-dimensional quantitative images related to interesting physicochemical properties of living cells [33,34]. PeakForce Tapping (PFT) in liquid media is a novel, cutting edge breakthrough in AFM that allows the imaging and quantification of the physicochemical properties associated to every point in a 3D surface immersed in a liquid environment. This is of special interest for biological samples and particularly for marine biofilms, so we have been able to measure these properties directly in natural seawater. In this article FD-AFM methods have been used to characterise the morphology of biofilms of *S. algae* grown in different nutritive media and to obtain quantitative mapping of elastic modulus and adhesion forces of the resulting biofilms.

## Results and discussion

### Influence of the culture conditions on bacterial growth and slime production

Bacterial growth was initially checked in agar plates of the nine culture media at 20°C, 26°C and 32°C after 24 h in order to qualitatively assess the best range of temperatures. From these initial observations, the lower incubation temperature was ruled out due to poor growth.

Media with different characteristics were chosen (Additional file 1: Table S1): Marine broth (MB) is a widespread culture medium for marine bacteria that contains high levels of salts as well as trace elements. Its main difference with the Supplemented Artificial Seawater medium (SASW) and Luria Marine Broth (LMB) is the amount of primary sources of carbon and nitrogen, and the trace element content [35]. Väätänen Nine-Salt Solution (VNSS) is a complex salt-rich medium that is frequently used in marine microbiology [36,37]. Mueller-Hinton is the standard culture medium in antimicrobial susceptibility tests, and often it needs to be supplemented with salts (2%, MH2) and/or calcium and magnesium (cation-adjusted MH2, CAMH2) to support the growth of certain bacteria like pathogenic vibrios [38,39] and halophilic marine strains [40,41]. Brain-Heart Infusion and Tryptic Soy Broth were also supplemented with 2% NaCl and designed as BHI2 and TSB2, respectively. These NaCl-supplemented rich media have been previously employed in the culture of *Pseudoalteromonas* and *Vibrio* species [15,16]. A minimal medium (MMM) was included to evaluate the effect of a limiting environment on biofilm formation. The actual starting cell density was 7.0 ± 0.8 × 10^5^ cfu/ml.

**Figure 2:**
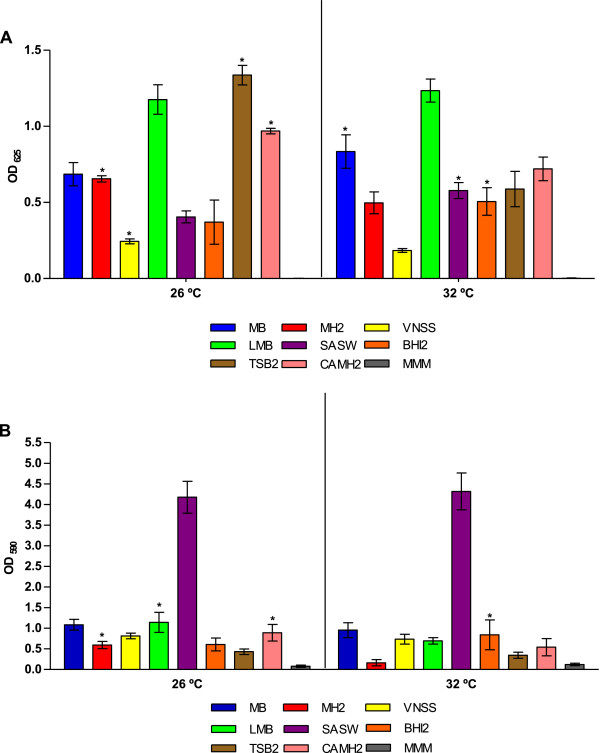
**Growth and biofilm formation under different conditions. (A)** Growth of *S. algae* CECT 5071 in different media after an incubation period of 24 h at 26°C and 32°C. **(B)** Biofilm formation under the same conditions. Bar heights represent the mean of eight replicates. Errors are expressed as ± standard deviation (SD). Asterisks indicate significant differences for Bonferroni post-hoc comparisons between both incubation temperatures for the growth medium, and they are shown above the bar with the highest OD in each case. OD values below 0.05 at any temperature have not been taken into account for statistical purposes.

Figure 2 shows the total cell density (A) and biofilm biomass (B) in different media at the two selected temperatures. In order to determine the effect of the medium, the temperature and the interaction on the total cell density and biofilm formation, ANOVA tests were performed. Without loss of generality for the goal of the study, optical density (OD) values below 0.05 have been considered as no total cell density/no biofilm formation and have not been taken into account for the ANOVAs purposes (Additional file 2: Table S2). From the results of the ANOVA tests, the culture medium, the incubation temperature and the interaction were highly significant for the total cell density and biofilm formation.

Clearly, rich media enhanced not only the growth but also biofilm production. However, higher total cell densities were not necessarily correlated with higher amounts of slime (Table 1). These rich media contain also high amounts of salts, particularly SASW, for which a marked shift in biofilm production is observed (Additional file 2: Table S2, Figure 2B). Indeed, salts increase protein adsorption on material surfaces, thus facilitating the establishment of the extracellular polymeric substances (EPS) that constitute the biofilm matrix [42]. Salt ions –cations, particularly-, are known to play important functions in the biofilm formation process of halophylic bacteria as their cytoplasmic enzymes are adapted to high ionic strengths and consequently they have a requirement of charged ions to function properly [43]. In this context, Ca^2+^ has been observed to be a requirement for the maintenance of biofilm integrity in certain variants of *Vibrio cholerae* and other marine Vibrio species [44], and a triggering factor of the synthesis of class II proteins in *Pseudoalteromonas* sp., which are expressed only in biofilm cells [45]. In *Pseudomonas fluorescens*, Mg^2+^ cation increase cell attachment and condition the structure and further development of the biofilms [46]. Cations such as Ca^2+^, Mn^2+^, Cu^2+^ or Zn^2+^ have also been found to be essential for the formation of air-liquid interface biofilms in *Shewanella oneidensis*[47]. In fact, when MH2 is supplemented with 20 mg/L Ca^2+^ and 10 mg/L Mn^2+^ (CAMH2 medium), a shift in biofilm production is observed (Figure 2B).

**Table 1:** Relative biofilm formation for each medium and temperature

**Culture medium**	***S*. *algae***	
	**26°C**	**32°C**
MB	1.58 ± 0.26	1.14 ± 0.26
MH2	0.91 ± 0.14	0.33 ± 0.16
CAMH2	0.92 ± 0.21	0.75 ± 0.30
BHI2	1.64 ± 0.77	1.66 ± 0.77
TSB2	0.32 ± 0.05	0.59 ± 0.17
LMB	0.97 ± 0.22	0.56 ± 0.07
SASW	8.60 ± 0.84	6.03 ± 0.55
VNSS	3.33 ± 0.36	3.99 ± 0.70
MMM	-	-

Values indicate the mean ± SD (n = 8) OD_590_/OD_625_ ratios. OD values below 0.05 were considered as no growth/no biofilm formation and have not been taken into account (indicated by a dash).

The temperature also exerted a significant influence on both growth and biofilm formation in function of the medium (Figure 2). The influence of the temperature on bacterial biofilm formation is usually species and strain-dependent. For example, Stauder *et al*. [48] positively correlated biofilm production and temperature in a *V. cholerae* strain, suggesting that higher seawater temperatures increase the persistence of the bacterium in the aquatic environment. Similarly, Chiu *et al*. [49] associated changes in the planktonic and biofilm bacterial communities with seasonal variations in water temperature and salinity. In a different study, McDougald *et al*. [50] found no correlation between temperature and biofilm formation in clinical and environmental strains of *Vibrio vulnificus*, whereas previous work showed a direct correlation between temperature, salinity and biofilm formation in the same bacterial species [51]. In that case, those findings were attributed to strain differences.

### The IC_50_ of model antifouling biocides on *Shewanella algae* is influenced by the culture medium and the starting cell density

There is clear evidence that the characteristics of the growth medium as well as the inoculum size may have a great influence on the results obtained from susceptibility tests [52-54]. To explore the effect of these two parameters, changes in the half-maximal inhibitory concentration (IC_50_) of three model biocides on *S. algae* were studied: the banned TBTO, a metal-based antifouling agent (zinc pyrithione) and a non-metal antifoulant (tralopyril). Three initial cell densities were employed: the standard inoculum size (S) prepared as described in the methods section as well as half and double this amount (H and D, respectively). Also, four growth media: MB, LMB, SASW and MH2 were selected. In these media *S. algae* presented different growth values and total biofilm production (Table 1).

Inoculum sizes were determined by plate counts (H = 3.5 ± 0.6 × 10^5^ cfu/ml, n = 4; S = 7.0 ± 0.8 × 10^5^ cfu/ml, n = 4; D = 1.5 ± 0.6 × 10^6^ cfu/ml, n = 4). The stock solutions of the biocides were prepared in dimethylsulfoxide (DMSO). The maximum percentage of DMSO inside a well was 0.25%. At this concentration, no growth inhibition was observed. Table 2 summarises the results obtained in this experiment.

**Table 2:** **IC**_**50 **_**values for three antifouling biocides towards *S. algae *** **CECT 5071**

**Culture medium**	**Inoculum size**	**IC**_**50 **_**(μM)**		
		**TBTO**	**Tralopyril**	**Zinc pyrithione**
MB	H	7.8 ± 1.3	14.6 ± 5.8	17.6 ± 1.1
	S	8.1 ± 1.5	15.8 ± 2.7	13.8 ± 2.0
	D	12.0 ± 2.3	19.9 ± 7.3	35.4 ± 6.1
MH2	H	10.7 ± 0.6	12.8 ± 3.5	12.8 ± 2.6
	S	10.3 ± 0.3	16.0 ± 1.8	18.9 ± 1.7
	D	12.4 ± 1.1	14.9 ± 3.4	16.7 ± 3.3
LMB	H	8.4 ± 0.5	1.9 ± 0.4	16.7 ± 2.5
	S	9.0 ± 0.3	2.5 ± 1.4	22.7 ± 6.5
	D	10.6 ± 1.4	2.0 ± 0.9	23.2 ± 6.6
SASW	H	9.5 ± 0.4	18.0 ± 1.9	6.0 ± 0.4
	S	11.4 ± 0.3	16.7 ± 0.9	7.8 ± 1.9
	D	12.8 ± 0.5	17.3 ± 1.6	7.8 ± 0.7

Data (mean ± SD, n = 3) are arranged in function of the culture medium and the initial cell density in each case. S = Standard inoculum; H = Half standard inoculum; D = Double standard inoculum.

The effect of the culture medium and the inoculum size on the IC_50_ were assessed by two-way ANOVAs. For TBTO, the culture medium and the inoculum size were highly significant, and no interaction was detected. For tralopyril only the culture medium was highly significant. Finally, for zinc pyrithione the culture medium, the inoculum size and the interaction were highly significant.

In spite of the diversity of conditions employed in bacterial antifouling bioassays in terms of inocula and media [5-11], the comparative effect of these conditions on the activity of model antifouling molecules has been poorly evaluated. The need for reproducible positive controls to validate the assays has been underlined previously [55]. Research in other areas with bacteria that require particular growth conditions such as lactic acid bacteria has highlighted the influence of the culture conditions on the activity of antibiotic standards [56,57]. The results obtained for *S. algae* show a dependence of the IC_50_ of antifouling biocides on small variations in the inoculum size and on the use of different culture media, which emphasizes the need for a consensus in this regard. A tempting alternative would be the adaptation of CLSI standards for antimicrobial susceptibility testing to the requirements of biofouling-representative bacteria. It is interesting to note that biofouling is a phenomenon of biological adhesion and consequently, growth inhibition may not be the main endpoint for biological assays [55]. Consequently, conditions a) supporting bacterial growth, b) promoting biofilm formation and c) mimicking a salt-rich environment would be desirable.

### *Shewanella algae* biofilms developed in different media exhibit medium-dependent morphological and nanomechanical properties

The final step in this study sought the answer for two questions: i) how is *S. algae* biofilm structure affected by the culture medium? and ii) how these different nutrient environments affect the mechanical properties of the biofilms? For this purpose, CLSM and AFM analysis were conducted on 24-hour *S. algae* biofilms developed in the four selected media. In order to respect exactly the same substrate as that employed for the initial *in vitro* experiments, the bottom of the wells of a microtiter plate were mechanically sectioned, sterilised, and used to develop the bacterial biofilms.

**Figure 3:**
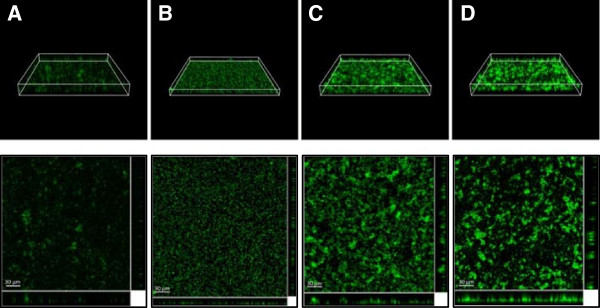
**Effect of the medium on biofilm structure evidenced by CLSM.** Projections (upper row) and sections (bottom row) of 24-h *S. algae* CECT 5071 biofilms (40x) developed in different media. Columns: **(A)** MB; **(B)** MH2; **(C)** LMB; **(D)** SASW.

CLSM analysis revealed significant differences in biofilm thickness, surface coverage and morphology (Table 3, Figure 3, Additional file 3: Figure S1 and Additional file 4: Table S3). *S. algae* biofilms reached almost 30 μm thick in SASW (Figure 3D, Additional file 3: Figure S1D). Similarly, biofilms developed in LMB and MB surpassed the 20 μm thick, even though the surface coverage was notably lower in MB (Figures 3A and C, Additional file 3: Figures S1A and C). A completely different structural pattern was observed in MH2. In this medium, *S. algae* developed comparatively thin biofilms, reaching a maximum of 13.5 μm, but exhibiting a comparatively high surface coverage (Figure 3B, Additional file 3: Figure S1B). Roughness coefficients are indicative of the degree of heterogeneity of the biofilms [58]. In fact, these values (Table 3), which are significantly different in function of the medium in which the biofilms were formed (Additional file 4: Table S3) agree with the visual evidence (Figure 3), and indicate a patchy, heterogeneous biofilm development in MB and SASW, and more uniform biofilm layers in MH2 and LMB.

**Table 3:** Average values of different biofilm properties in the four selected media

**Medium**	**Mean thickness (μm)**	**Max. thickness (μm)**	**Coverage (%)**	**Roughness coefficient**	**Young modulus (MPa)**	**Adhesion (nN)**
MB	11.2 ± 0.8	25.3 ± 2.3	15.9 ± 1.7	1.92 ± 0.06	0.16 ± 0.10	1.33 ± 0.38
MH2	9.0 ± 1.2	13.5 ± 1.0	20.9 ± 2.4	0.97 ± 0.15	0.34 ± 0.16	0.73 ± 0.29
LMB	15.4 ± 2.2	20.5 ± 3.4	32.1 ± 4.6	0.65 ± 0.18	0.22 ± 0.13	0.85 ± 0.35
SASW	13.0 ± 0.8	29.5 ± 1.9	23.9 ± 3.9	1.40 ± 0.24	0.19 ± 0.09	1.11 ± 0.41

Biofilm thickness (n = 12), surface coverage (n = 12) and roughness coefficients (n = 12) were determined from CLSM reconstructions. Young modulii and adhesion forces were quantified by AFM. In this case, at least 115 bacteria were individually analysed for each magnitude. Data represent the average ± SD.

Thus, two trends were observed in biofilm development depending on the medium: a clear trend to a three-dimensional growth, with a variable degree of homogeneity, in MB, LMB and SASW, and a relatively horizontal development in MH2, maximising cell-to-cell and cell-to-substrate interactions. According to this depiction, we will focus on the comparison between MB and MH2 since they have been considered representative enough of the two biofilm growth behaviours.

First of all, in order to show the topographic features exhibited by the studied cells at high resolution, the samples were imaged in air after being rinsed and dried. Thus, Figure 1A shows a representative picture of some *S. algae* cells attached to the treated polystyrene substrate. Since these images were obtained in air, some flagella belonging to neighbouring bacteria adsorbed on the surface could be observed as well. Bacterial cells were 2.2-3.5 μm in length and 0.4-0.7 μm in width. Some polishing lines resulting from the disc’s surface treatment are also visible. Additionally, in Figure 1B, some of these features can be observed in more detail, namely some flagella (white arrow), topographic details of the bacterial surface and submicrometer particles of EPS.

**Figure 4:**
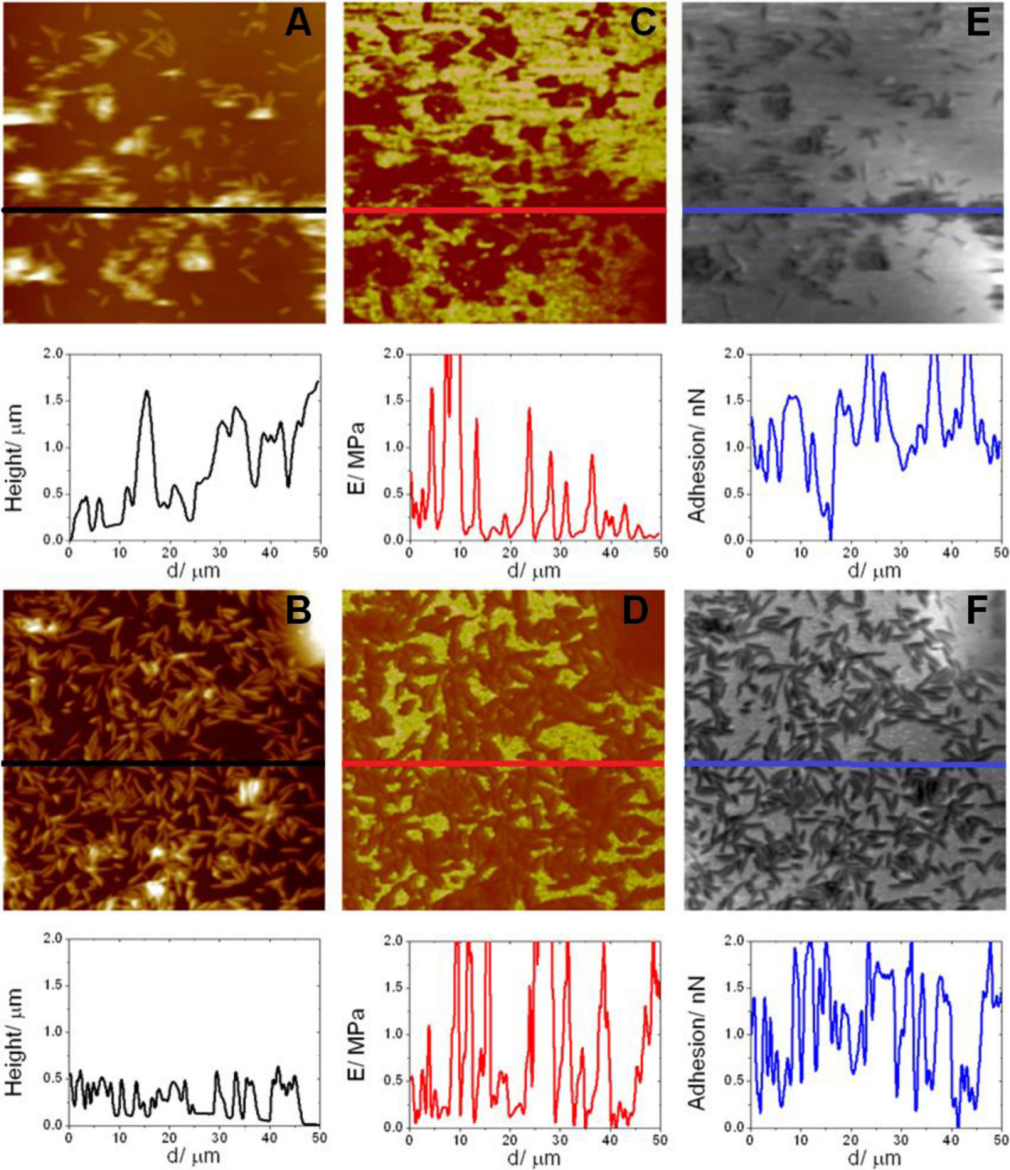
**Representative cross-section of 2D peak force tapping 50 x 50 μm**^**2 **^**images. (A)** and **(B)**, topographic images of MB and MH2, respectively, in brown; **(C)** and **(D)**, Young’s modulus quantitative, in gold; **(E)** and **(F)**, adhesion forces, grey.

Figures 4A-B correspond to AFM topographic images recorded in 0.22 μm filtered seawater (FSW) obtained in MB and MH2, respectively. Nevertheless, supplementary information (Additional file 5: Figures S2, Additional file 6: Figure S3, Additional file 7: Figure S4, Additional file 8: Figure S5, Additional file 9: Figure S6 and Additional file 10: Figure S7) contains the AFM results obtained for all the media studied. Three-dimensional aggregates formed by bacteria linked to each other can be seen in MB, leaving large bacteria-free areas. Conversely, in Figure 4B, the substrate appears to be covered by a near continuous and homogenous layer of bacteria and EPS. In this case, three-dimensional aggregates are present in a remarkably lower degree. These results revealed a different interaction between the substrate and the bacterial envelope in function of the culture medium. Thus, in MH2, bacteria-substrate interaction is clearly favoured in comparison to MB.

On the other hand, Figures 4C-D compare the Young’s modulus and Figures 2E-F the adhesion force quantitative mappings of the same surface area for MB and MH2. In this context, it should be taken into account that the greater the brightness of the patches the larger corresponding values of the magnitudes analysed. In general terms, images show that the higher values in Young’s modulus and adhesion force correspond to the bacteria-free substrate areas. Note that the higher pikes present in the cross sections (E > 0.7 MPa) are related to contributions due to bacteria/EPS-free substrate. Thus, Young’s modulus exhibited by bacteria resulted to be significantly larger for those grown in MH2 (Additional file 4: Table S3). However, regarding adhesion forces, the situation was exactly the opposite with the higher figures corresponding to MB. In addition, by considering the average size of certain Young’s modulus spots, especially those associated with clusters of bacteria present in the topographic image, it can be concluded that these groups of bacteria seem to be surrounded by EPS which spreads to the cell-substrate interface (see also Additional file 6: Figure S3A-F).

Table 3 shows the averaged values of Young’s modulus and adhesion forces recorded for individual bacterial cells grown in the four different media. Our overall experimental data (see histograms in Additional file 8: Figure S5) confirmed the trend previously described a clear correlation between the rising in Young’s modulus and the diminishing in the adhesion response is exhibited when modifying the growth medium. As shown in Table 3, values registered for MH2 almost doubled those grabbed for MB. Anyway, the biofilm developed in MH2 showed the highest elasticity values registered. It should be noted that these results obtained for the elasticity properties of the external covering layer of *S. algae cells* are in the same order of magnitude as those reported for other gram-negative bacteria [59,60]. In fact, significant variations in nanomechanical and physicochemical properties have also been reported for *Escherichia coli* and *S. putrefaciens* cells depending on the culture conditions and on the pH, respectively [61,62].

Average adhesion forces are shown in Table 3. As discussed before, an opposite correlation among data for Young’s modulii is observed. Thus, figures obtained for MH2 were significantly lower than those obtained for MB (Additional file 4: Table S3). Regarding this point, it should be noted that whereas Young’s modulus is directly dependent on the mechanical behaviour of the outer part of the bacteria under tip indentation, adhesion forces imply specific attractive interactions with the tip. In this sense, it is worth noting that the abovementioned correlation has not necessarily to be like that.

Although AFM tips have not been functionalised and consequently the adhesion force response recorded is due to non-specific interactions [63], it should be noted that AFM tips, bacteria and incubation times remained unchanged in all the experiences carried out. Therefore, differences observed for the biofilms grown in the different media reflect unambiguously a significant impact on the physicochemical properties of biofilms. Consequently, these results allow us to conclude the substantial effect of modifying the culture medium on the nanomechanical and physicochemical behaviour exhibited by the resulting biofilms.

**Figure 5:**
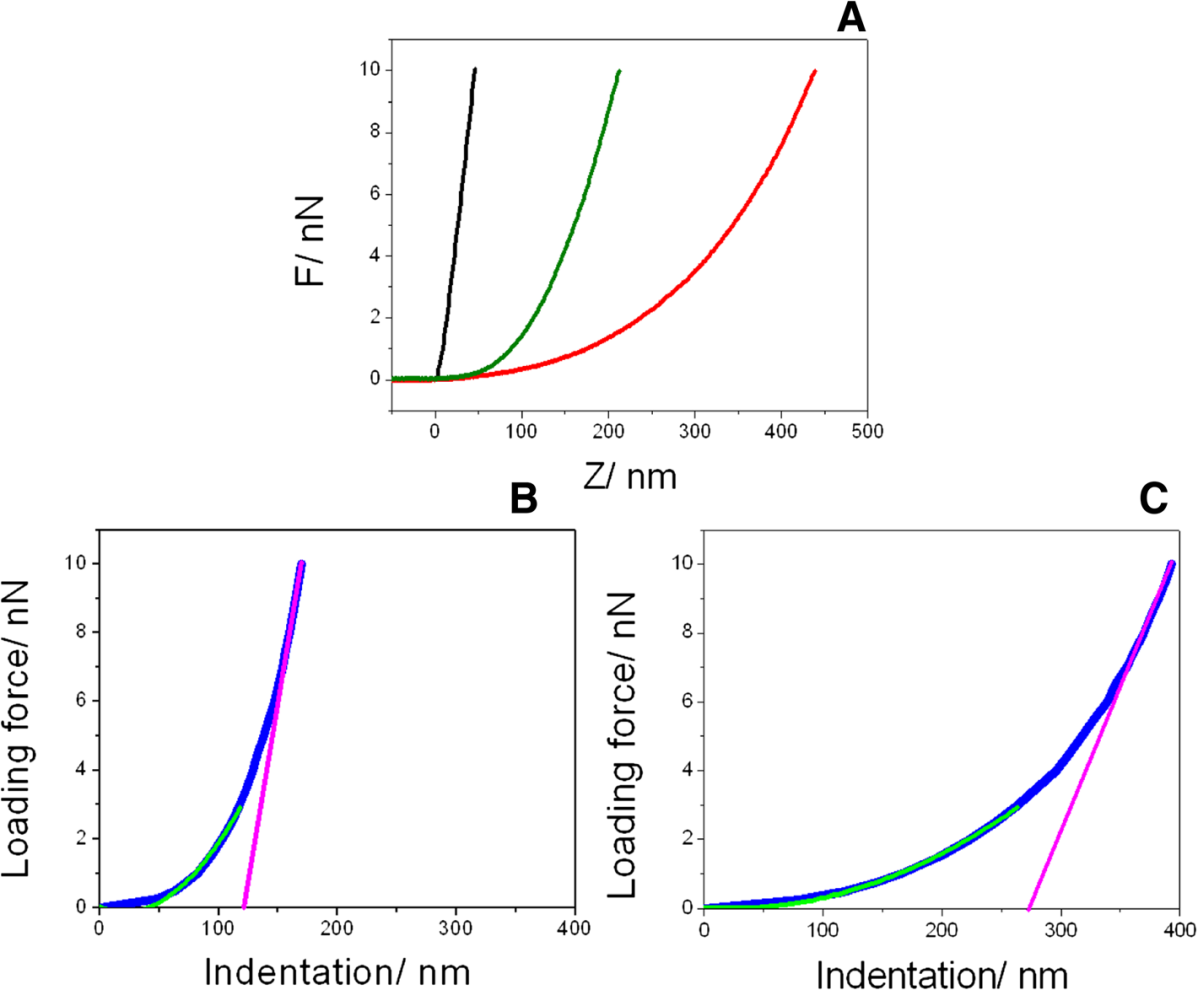
**Representative force-distance curves. (A)** Representative force-piezo displacement measured on mica (black) and on top of single bacteria grown in MH2 (dark green) and in MB (red), obtained in seawater. Loading force-indentation depth (blue) curves resulted from subtracting black curve from the green **(B)** and the red ones **(C)** at constant loading force. Curves **(B)** and **(C)** were fitted according Hertz’s model (green) and linear model (magenta) to calculate elasticity modulus and k_B_, respectively.

AFM force-distance curve analysis has also been carried out in order to assess k_B_, the spring constant of the bacteria, which eventually resulted also dependent on the growth medium. Thus, Figure 5A shows representative force-distance curves registered in seawater for a stiff surface, mica (black line), and for representative single deformable bacteria grown in MB (red) and in MH2 (dark green). In this context, considering the relevant differences exhibited by the indentation depths grabbed for MB and MH2, a differential elasticity response can be easily concluded. Indeed, envelope belonging to bacteria grown in MH2 showed noticeable more rigid profiles than those corresponding to MB (Figures 5B-C).

By combining properly the Hertz’s model and Hooke’s law, nanomechanical properties of the bacterial envelope can be deduced from the experimental loading force-indentation curves. Thus, according to the Hertzian model that takes into account the geometry of the tip, the non-linear regime corresponding to the initial part of the indentation curve was fitted to evaluate the Young’s modulus of the bacteria indented [62], green lines in Figures 5B-C. Likewise, from the linear part of the curve obtained from experimental data registered at high loading forces, the k_B_ of the bacteria could be calculated by linear fitting (magenta lines in Figures 5B-C) [59]. Elasticity values obtained were 0.15 ± 0.08 MPa for MB and 0.38 ± 0.11 MPa for MH2. As expected, Young’s modulus data resulted to be in very good agreement with those previously obtained by PF-QNM (Table 3). On the other hand, k_B_ values, which ranged from 0.022 N/m to 0.050 N/m, are consistent with those obtained for other gram negative bacteria as thoroughly reported [59,61]. Moreover, these figures exhibited the same trend showed by elastic modulus when altering the culture medium.

## Conclusions

The influence of the culture medium and the incubation temperature on the total cell density and biofilm formation of *Shewanella algae* CECT 5071 has been studied. The influence of both factors was found to be highly significant. Additionally, the culture medium and the inoculum size exerted a significant influence on the values obtained for the IC_50_ of three antifouling biocides. An approach to the unification of criteria in antifouling bioassays involving marine bacteria could be the adaptation of already existing, universally-accepted methodologies to the requirements of test organisms concerning marine biofouling. With regard to bacteria, CLSI guidelines constitute the most evident and clear reference.

With this work we have established and characterised in detail a biofilm model for antifouling bioassays. Using *S. algae* CECT 5071 as model organism, we were able to demonstrate quantitatively the influence that the culture medium exerts not only on the biofilm density or thickness, but more importantly, on the biofilm structure and on its nanomechanical and physicochemical properties. CLSM showed two clear architectural patterns in function of the medium in which the biofilms were developed. From PF-QNM and FD-AFM data it is possible to infer that *S. algae* cells grown in MH2 medium exhibited a more complex outer surface, remarkably stiffer and with a significant higher range of Young’s modulus figures distribution, when compared to the other media which showed more similar features in this sense. On the other hand, adhesion forces results evolved in the opposite way thus confirming the differential physicochemical behaviour exhibited by the biofilms in function of the nutrient environment.

## Methods

### Strains and assay platform

*Shewanella algae* CECT 5071 was acquired from the Spanish Type Culture Collection (CECT). The strain was cryopreserved at −80°C. Before each experiment, an agar plate was streaked and incubated for 24 h. A single, isolated colony was selected to streak a second agar plate that was incubated for other 24 h. Inocula were prepared from these second agar plates. The experiments were conducted in 96-well flat-bottom surface-treated polystyrene microtiter plates (Nunc 167008). For biofilm experiments, the bottom of the wells were used as substrate (see Microscopy: general procedures section).

### Culture media

Bacterial growth and biofilm formation were quantified in nine different media: Marine Broth (MB) (Conda); Mueller-Hinton Broth (Scharlau) supplemented with NaCl to give a final concentration of 2% (MH2); cation-adjusted MH2 (CAMH2), that consisted in MH2 supplemented with 55 mg/l CaCl_2_ and 40 mg/l MgCl_2_; Brain Heart Infusion (Scharlau) supplemented with NaCl to a final concentration of 2% (BHI2); Tryptic Soy Broth (BD) supplemented with NaCl to a final concentration of 2% (TSB2); Luria Marine Broth (LMB); Supplemented Artificial Seawater (SASW); Väätänen Nine-Salt Solution (VNSS); and Marine Minimal Medium (MMM). LMB and SASW were prepared according to Lang *et al*. [35], NSS and VNSS followed the recipe described by Mårdén *et al*. [64]; and MMM was prepared as described by Östling *et al*. [65]. A summary of the composition of each medium is provided as additional information (Additional file 1: Table S1).

### Assessment of growth and biofilm production

Each well of the microtiter plate contained 100 μl of bacterial inoculum and 100 μl the appropriate culture medium. Growth at two temperatures (26 and 32°C) was quantified after an incubation period of 24 h by measuring the optical density at 625 nm (OD _625_) with an automatic plate reader (Perkin-Elmer EnSpire). Eight replicates were used for each medium.

Once the growth was measured, biomass was quantified by the crystal violet (CV) staining method [66]. Briefly, wells were thoroughly washed three times with water to remove the culture medium and planktonic cells as well as loosely adhered bacteria. Firmly attached bacteria were heat fixed (65°C) for 30–45 min and then 200 μL of a 0.2% CV solution (Sigma-Aldrich) were added to each well. After 15 min wells were emptied and washed carefully with water. Plates were air-dried and then the dye was solubilised by addition of 200 μl of absolute ethanol. Absorbance was recorded at 590 nm. When OD_590_ readings were above 2.5, the sample was tenfold diluted and OD was measured again [67].

Three classic antifouling agents: TBTO, tralopyril and zinc pyrithione were purchased from Sigma-Aldrich. Stock solutions of the products (40 mM) dissolved in dimethylsulfoxide (DMSO) were diluted in the culture medium to give a final test concentration of 100 μM. Serial dilutions (100, 50, 10, 5, 1, 0.5, 0.1 and 0.05 μM) were performed for the determination of the IC_50_ in MB, MH2, LMB and SASW. OD readings were normalised with respect to the absorbance of the blank wells and then the growth inhibition percentage respect to a control with the proportional amount of DMSO was calculated. Experiments were run by triplicate.

### Preparation of inocula

Bacterial inocula were prepared in 0.22 μm filtered seawater (FSW). Isolated colonies were suspended until they matched a McFarland turbidity of 0.5 (bioMérieux Vitek Densichek). One hundred microliters were transferred to test tubes containing 9.9 ml of the appropriate culture medium. This cell suspension constituted the standard starting inoculum (S) as defined by CLSI guidelines for antimicrobial susceptibility testing [68]. Double (D) and half (H) the size of the standard inoculum were used to evaluate the effect of the initial cell density on the activity of biocides towards *S. algae*. To check the actual starting cell number, a 200 μl sample of the inoculum was serially tenfold diluted from 10^−1^ to 10^−8^. Four 10 μl drops from each dilution were spotted on agar plates and incubated. Colony formation was assessed after 24 h.

### Microscopy: general procedures

For microscopy experiments, the bottoms of the wells of a microtiter plate were mechanically sectioned with a computer numerical control milling machine (Fagor CNC 8055 M) in order to use exactly the same substrate as in previous tests. The sectioned discs thus obtained (5.86-5.98 mm in diameter, 1.00-1.08 mm in height, data from 15 random measurements) were carefully disengaged and sterilised by a brief sonication in ethanol and UV irradiation before their use in the experiments. To develop the biofilms, the discs were placed at the bottom of a 24-well microtiter plate. Two-mililiter bacterial cultures were prepared in the appropriate medium following the same procedures as described previously. After the incubation period, discs were rinsed three times with FSW and kept immersed upon their use in the microscope.

### Confocal Laser Scanning Microscopy

Biofilms formed on polystyrene discs were fluorescently stained with acridine orange (AO), a membrane permeant nucleic acid stain that intercalates dsDNA and binds to ssDNA as well as to ssRNA through dye-base stacking to give broad spectrum fluorescence when excited at 476 nm [69]*.* This compound stains all cells in a biofilm, live or dead, and may also bind to nucleic acids that are present in the extracellular matrix. To stain biofilms, discs were immersed in 0.1% w/v AO (Sigma-Aldrich) in PBS for 5 min at room temperature and washed with FSW. Fluorescently labelled biofilms were placed in two drops of 0.9% FSW on the surface of a glass coverslip and were examined using an Olympus Fluoview 1000 Confocal Laser Scanning Microscope. Each biofilm was scanned at 4 positions randomly selected at the microscope stage and confocal image series were generated by optical sectioning at each of these positions. Three independent biofilm experiments were performed, and image stacks of 512×512 pixels were collected for quantification. Image combining and processing were performed with the Imaris software package, version 4.0 (Bitplane AG, Zürich, Switzerland). The biofilm structure was quantified using the software program COMSTAT [70] available as free downloadable software at http://www.imageanalysis.dk. COMSTAT converts pixels from confocal image stacks into numerical values, facilitating quantitative characterization of each structural component within 3D biofilm images [71]. COMSTAT software allowed the determination of:

– Mean thickness: the average thickness, from the base at the biofilm–substratum interface to the top of the biofilm in the channel lumen, across the entire biofilm in the field of view. Mean biofilm thickness provides a measure of the spatial size of the biofilm.

– Maximum thickness: the maximum thickness over a given location, ignoring pores and voids inside the biofilm.

– Roughness coefficient: a measure of variation in biofilm thickness across the field of view, an indicator of biofilm heterogeneity.

The percentage of adhering cells (% Coverage) was calculated using ImageJ NIH image processing software [72].

### Atomic Force Microscopy

Imaging and force measurements to characterise the nanomechanical properties of *Shewanella algae* cells were performed by AFM. In these studies every treated polystyrene disc containing the immobilised bacteria was attached to a steel sample puck by means of an adhesive tape. When measuring in liquid, 50 μL of FSW were added onto the disc prior to be placed into the AFM liquid cell. For measurements performed in air, polystyrene discs were carefully rinsed and dried in N_2_ atmosphere before using.

Tapping Mode: *S. algae* cells were imaged by AFM operating in tapping mode in air using a Multimode microscope and a Nanoscope V control unit from Bruker at a scan rate of 1.0–1.2 Hz. To this end, etched silicon tips (RTESP, 271–311 kHz, and 40–80 N/m) were used.

Peak Force Tapping and force-distance analysis: Quantitative mapping were performed in FSW at room temperature using a Nanoscope V controller (Bruker). Images were acquired in AFM contact and Peak Force Tapping Mode [73] (Peak Force-Quantitative Nanomechanics, PF-QNM). AFM probes used in these studies were silicon nitride probes (NP-C, Bruker) with a nominal tip radius of 20–60 nm. The spring constant of cantilevers were measured using the thermal tuning method [74], and its values ranged 0.14-0.26 N/m. Mica surfaces were selected as rigid substrates for deflection sensitivity calibration. Note that in PF-QNM measurements AFM tips were carefully calibrated before every experience as described elsewhere [74-77]. Experimental results were acquired for single bacteria or little groups of them from the PF-QNM images, excluding thus contributions due to bacteria/EPS-free substrate. Data proceeding from at least 115 units from two independent cultures were collected for each medium. Adhesion force and Young’s modulus values distribution has been expressed as histograms. Force-distance (FD) curves were collected using low loading forces (F < 20 nN) in order to protect both the AFM tip and the bacterial cells [59]. Data processing was carried out using the commercial Nanoscope Analysis (Bruker), WSxM (Nanotec) [78] and Gwyddeon (GNU) softwares.

### Statistics

The effects of culture medium, incubation temperature and their interaction on the dependent variables (total cell density and biofilm formation) were assessed by a two-way ANOVA. The effects of culture medium, the inoculum size and their interaction on the IC_50_ were assessed by two-way ANOVAs for different biocides with *S. algae*. In all cases a balanced design was performed and a fixed-effects model of analysis of variance was applied. In some cases the response variable was square root transformed to improve homocedasticidy. Bartlett test was performed to check this assumption and normality was verified by means of Kolmogorov-Smirnoff test for residuals. Tukey or Bonferroni multiple comparison post hoc tests were assessed in all the instances. The IBM® SPSS® Statistics 19.0 was used for statistical analysis. A significance level at 0.05 was set.

To assess the effect of culture medium on *S. algae* biofilm structure, a one way ANOVA for each of the following variables: mean and maximum thickness, coverage and roughness coefficient, were performed, followed by a Tukey test to check for differences between the four culture media. Mean thickness was logarithmic transformed to improve homocedasticity. Moreover, the effect of culture medium on the Young’s modulus and adhesion force, both of them normally distributed but with unequal variances, was conducted by means of a Welch one-way ANOVA followed by a Games Howell post hoc test. For all the variables, the culture medium was highly significant.

Half-maximal inhibitory concentration values (IC_50_) were determined with GraphPad Prism 5 using a four-parameter non-linear regression model (GraphPad Software Inc., La Jolla, CA, USA).

## Competing interests

The authors declare that they have no competing interests.

## Authors’ contributions

AJM-R and JJF conceived and designed the experiments. AJM-R conducted the experiments. AG-O and AH-C conducted the AFM work and processed the results from AFM measurements. AM conducted the CLSM work. RD-G carried out the statistical analysis. VSM and MN contributed with reagents, materials and valuable advice in the experimental design. AJM-R, AG-O, AH-C and JJF analysed the data. AJM-R and JJF wrote the paper. All authors read and approved the final manuscript.

## Supplementary Material

Additional file 1: Table S1 Media composition. A detailed list of the components of each medium is provided (g/l). Click here for file

Additional file 2: Table S2 Two-way ANOVA test design and results for the growth and biofilm formation experiments. Two-way ANOVA was conducted with total cell density and biofilm formation as dependent variables and two factors, culture medium and incubation temperature. The dependent variable has been square-root (SR) transformed to ensure homocedasticity. Click here for file

Additional file 3: Figure S1 Detail of biofilm thickness in each medium. (A) MB; (B) MH2; (C) LMB; (D) SASW. Click here for file

Additional file 4: Table S3 One-way ANOVA and Welch ANOVA results for CLSM and AFM data, respectively. For the one-way ANOVA, the dependent variable has been logarithmic transformed to ensure homocedasticity. Click here for file

Additional file 5: Figure S2 Representative 3D Peak Force Tapping 50 x 50 μm^2^ images of *Shewanella algae* grown in different nutritive media. (A) MB; (B) MH2; (C) LMB; and (D) SASW. Click here for file

Additional file 6: Figure S3 Representative 3D Peak Force Tapping 50 x 50 μm^2^ images (A)-(D), topographic images corresponding to media MB, MH2, LMB, and SASW, respectively, in brown; (E)-(H), Young’s modulus quantitative mappings, in gold; (I)-(L), adhesion forces, grey. Click here for file

Additional file 7: Figure S4 Representative cross-sections of 2D Peak Force Tapping 50 x 50 μm^2^ images. (A) and (B), topographic images of media LMB and SASW, respectively, in brown; (C) and (D), Young’s modulus quantitative mappings, in gold; (E) and (F), adhesion forces, grey*.* Click here for file

Additional file 8: Figure S5 Histograms showing the elastic modulus (E, red bars) and adhesion force (blue) distributions for *Shewanella algae* cells. (A) and (E) MB; (B) and (F) MH2; (C) and (G) LMB; (D) and (H) SASW. Click here for file

Additional file 9: Figure S6 Representative cross-section of 2D Peak Force Tapping 15 x 15 μm^2^ images. (A)-(B), topographic images of media MB, MH2, LMB, and SASW, respectively, in brown; (E)-(H), Young’s modulus quantitative mappings, in gold; (I)-(L), adhesion forces, grey. Click here for file

Additional file 10: Figure S7 Representative 2D Peak Force Tapping 2.7 x 2.7 μm^2^ (upper panel) and 4.5 x 4.5 μm^2^ (lower panel) images. (A) and (B), topographic images of media MB and MH2, respectively, in brown; (C) and (B), Young’s modulus quantitative, in gold; (E) and (F), adhesion forces, grey. Click here for file
