# Supplementary material for: On the influence of the culture conditions in bacterial antifouling bioassays and biofilm properties: Shewanella algae, a case study
Source: BMC Microbiol. 2014 Apr 23;14:102. doi: 10.1186/1471-2180-14-102 (PMC4021068; doi:10.1186/1471-2180-14-102)
Supplement: Additional file 1: Table S1 — Media composition. A detailed list of the components of each medium is provided (g/l). [file 1471-2180-14-102-S1.docx]

|  | **Medium** | | | | | | | | |
| --- | --- | --- | --- | --- | --- | --- | --- | --- | --- |
| **Components** | **MB** | **MH2** | **CAMH2** | **BHI2** | **TSB2** | **LMB** | **SASW** | **VNSS** | **MMM** |
| Amonium chloride |  |  |  |  |  |  | 5.00 |  | 0.51 |
| Amonium nitrate | 0.0016 |  |  |  |  | 0.0016 |  |  |  |
| Bacto^TM^ Soytone |  |  |  |  | 3.00 |  |  |  |  |
| Bacto^TM^ Tryptone |  |  |  |  | 17.00 |  |  |  |  |
| Beef infusion solids |  | 2.00 | 2.00 |  |  |  |  |  |  |
| Boric acid | 0.022 |  |  |  |  |  |  | 0.008 | 0.008 |
| Brain extract |  |  |  | 12.50 |  |  |  |  |  |
| Calcium chloride | 1.80 |  | 0.055 |  |  | 1.80 | 1.47 | 0.41 | 0.41 |
| Casein hydrolisate |  | 17.50 | 17.50 |  |  |  |  |  |  |
| Dextrose |  |  |  | 2.00 | 2.50 |  |  | 0.50 | 0.40 |
| Dipotassium phosphate |  |  |  |  | 2.00 |  |  |  | 0.23 |
| Disodium phosphate | 0.008 |  |  | 2.50 |  | 0.008 | 0.89 | 0.01 |  |
| Ferric citrate | 0.10 |  |  |  |  | 0.10 |  |  |  |
| Ferric sulphate |  |  |  |  |  |  | 0.03 |  |  |
| Ferrous sulphate |  |  |  |  |  |  |  | 0.01 | 0.003 |
| Heart extract |  |  |  | 5.00 |  |  |  |  |  |
| Magnesium chloride | 8.80 |  | 0.040 |  |  | 5.90 | 5.08 | 1.87 | 1.89 |
| Magnesium sulphate |  |  |  |  |  |  | 6.16 |  |  |
| MOPS |  |  |  |  |  |  |  |  | 8.37 |
| Peptone | 5.00 |  |  | 10.00 |  | 10.00 | 3.50 | 1.00 |  |
| Potassium bromide | 0.08 |  |  |  |  |  |  | 0.04 | 0.04 |
| Potassium chloride | 0.55 |  |  |  |  | 0.55 | 0.75 | 0.25 | 0.25 |
| Sodium bicarbonate | 0.16 |  |  |  |  | 0.16 |  | 0.08 | 0.08 |
| Sodium chloride | 19.40 | 20.00 | 20.00 | 20.00 | 20.00 | 19.45 | 23.00 | 17.60 | 17.81 |
| Sodium fluoride | 0.0024 |  |  |  |  |  |  |  |  |
| Sodium silicate | 0.004 |  |  |  |  |  |  |  |  |
| Sodium sulphate | 3.24 |  |  |  |  | 3.24 | 1.47 |  | 1.49 |
| Starch |  | 1.50 | 1.50 |  |  |  |  | 0.50 |  |
| Strontium chloride | 0.034 |  |  |  |  |  |  | 0.008 | 0.008 |
| Tricine |  |  |  |  |  |  |  |  | 0.72 |
| Yeast extract | 1.00 |  |  |  |  | 5.00 | 3.50 | 0.50 |  |
