# Supplementary material for: On the influence of the culture conditions in bacterial antifouling bioassays and biofilm properties: Shewanella algae, a case study
Source: BMC Microbiol. 2014 Apr 23;14:102. doi: 10.1186/1471-2180-14-102 (PMC4021068; doi:10.1186/1471-2180-14-102)
Supplement: Additional file 2: Table S2 — Two-way ANOVA test design and results for the growth and biofilm formation experiments. Two-way ANOVA was conducted with total cell density and biofilm formation as dependent variables and two factors, culture medium and incubation temperature. The dependent variable has been square-root (SR) transformed to ensure homocedasticity. [file 1471-2180-14-102-S2.docx]

| Bacterial strain | Transform | Treatment | Total cell density | | Biofilm formation | |
| --- | --- | --- | --- | --- | --- | --- |
|  |  |  | **Levels for factor culture medium** | **Significance** | **Levels for factor culture medium** | **Significance** |
| *S*. *algae*  CECT 5071 | Total cell density: SR  Biofilm: SR | Medium  Temperature  Interaction | MB, MH2, CAMH2, BHI2, TSB2, LMB, SASW, VNSS | 0.000  0.000  0.000 | MB, MH2, CAMH2, BHI2, TSB2, LMB, SASW, VNSS, MMM | 0.000  0.000  0.000 |
