# Supplementary figures and images for: On the influence of the culture conditions in bacterial antifouling bioassays and biofilm properties: Shewanella algae, a case study
Source: BMC Microbiol. 2014 Apr 23;14:102. doi: 10.1186/1471-2180-14-102 (PMC4021068; doi:10.1186/1471-2180-14-102)

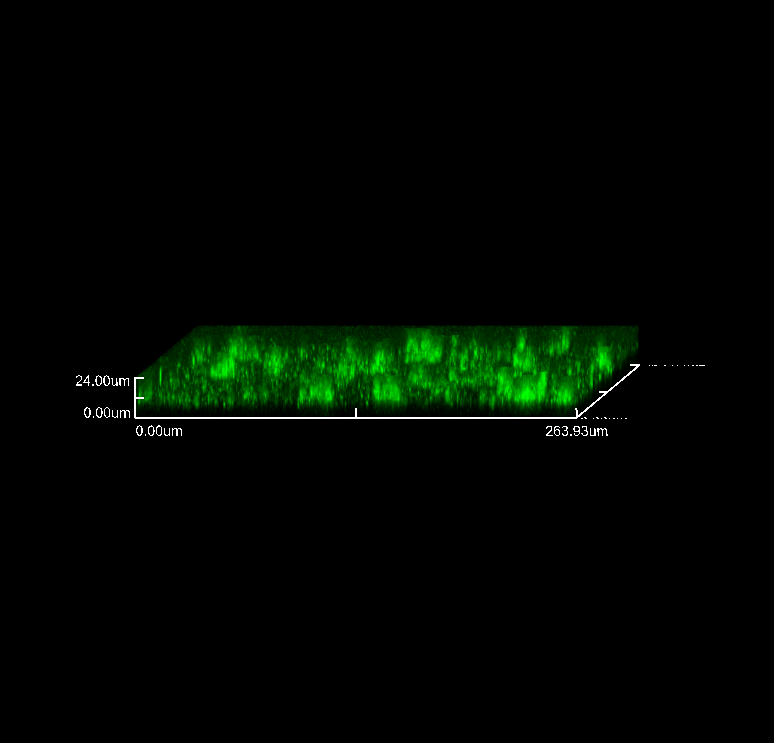


**A**


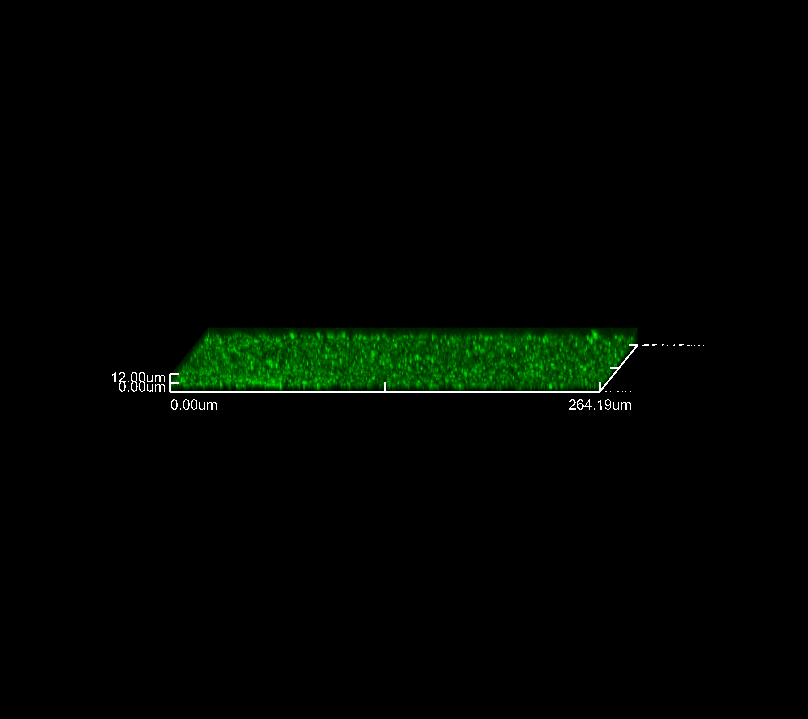


**B**


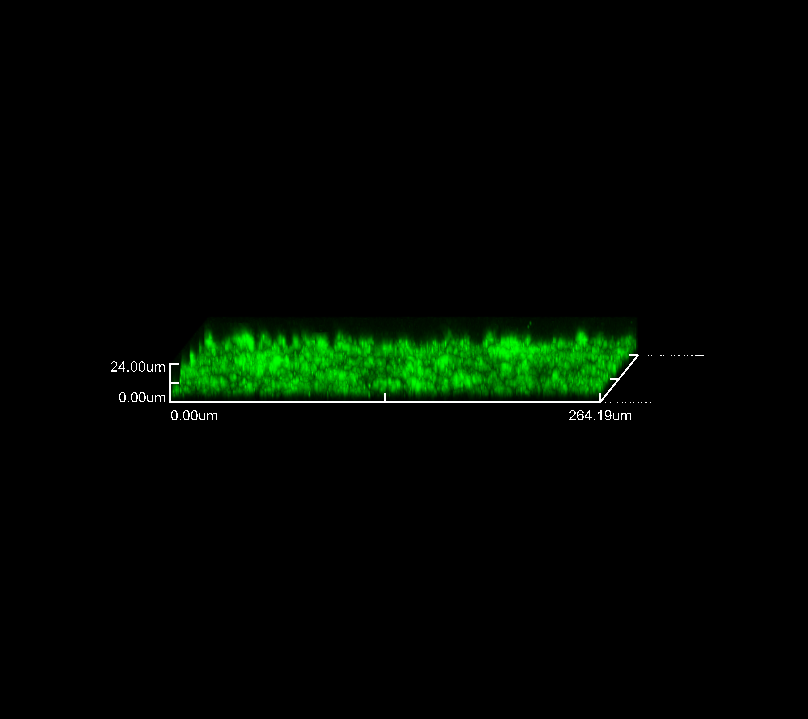


**C**


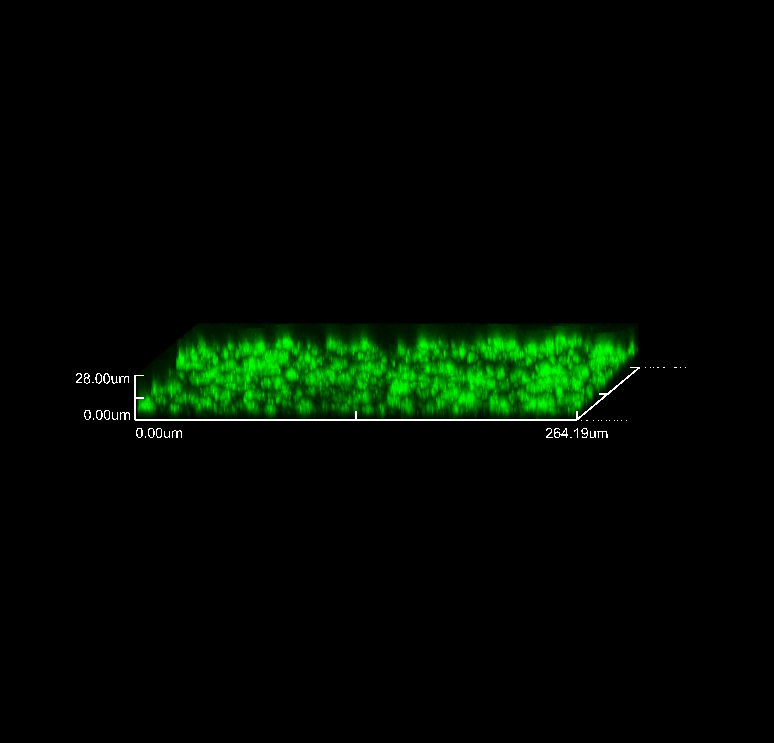


**D**

Supplement: Additional file 3: Figure S1 — Detail of biofilm thickness in each medium. (A) MB; (B) MH2; (C) LMB; (D) SASW. [file 1471-2180-14-102-S3.docx]

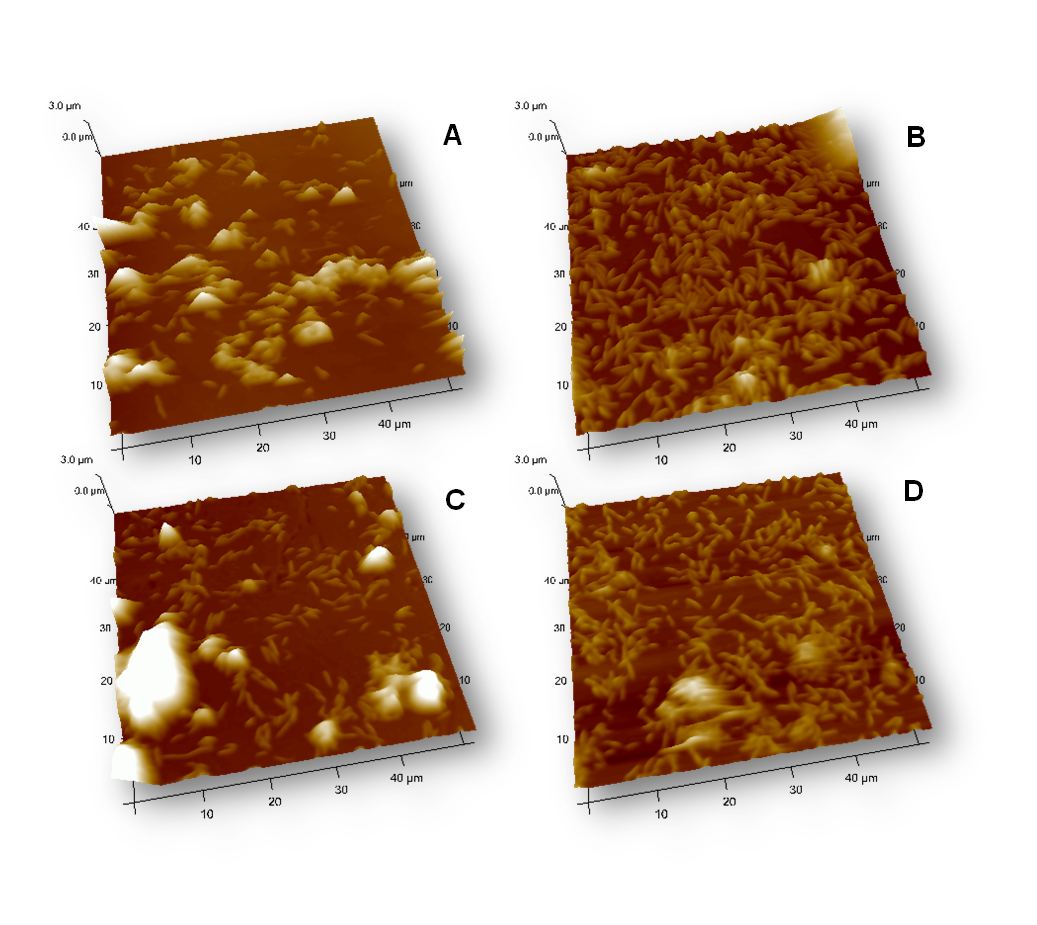

Supplement: Additional file 5: Figure S2 — Representative 3D Peak Force Tapping 50 x 50 μm2 images of Shewanella algae grown in different nutritive media. (A) MB; (B) MH2; (C) LMB; and (D) SASW. [file 1471-2180-14-102-S5.docx]

*
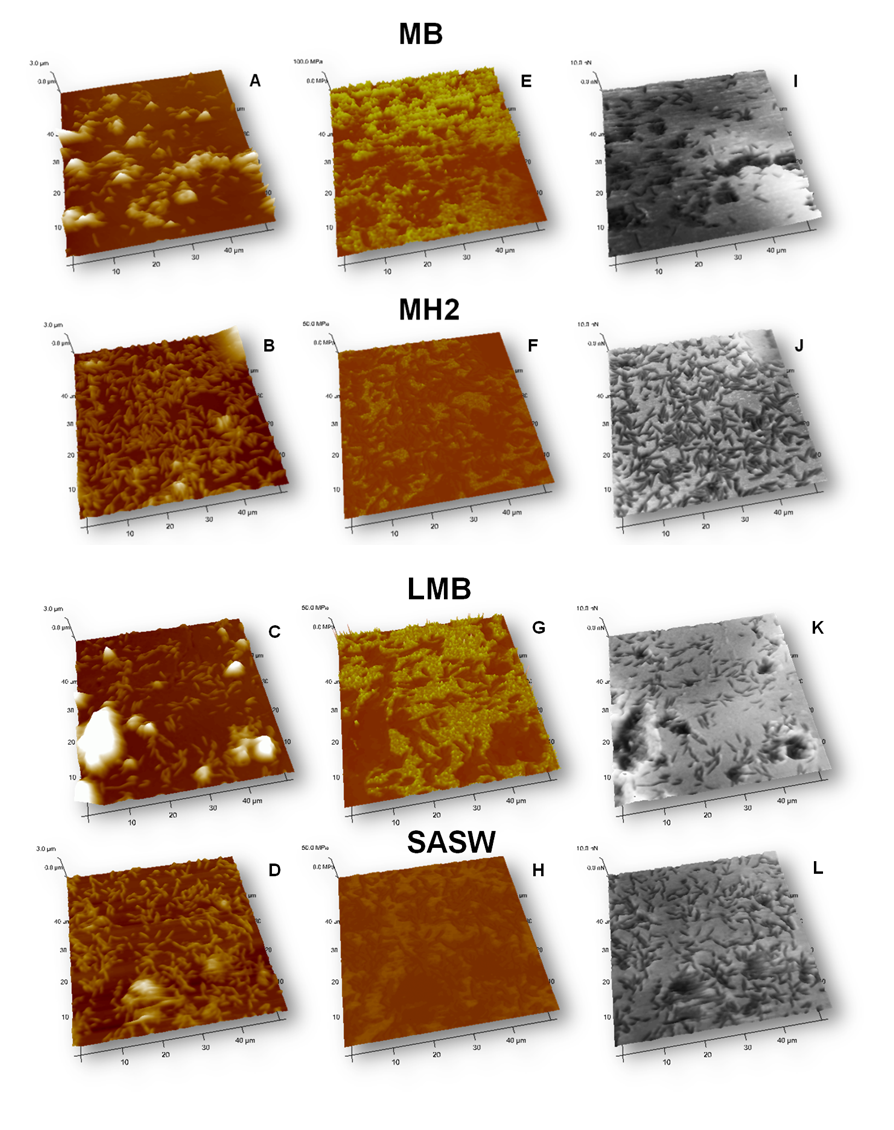
*

Supplement: Additional file 6: Figure S3 — Representative 3D Peak Force Tapping 50 x 50 μm2 images (A)-(D), topographic images corresponding to media MB, MH2, LMB, and SASW, respectively, in brown; (E)-(H), Young’s modulus quantitative mappings, in gold; (I)-(L), adhesion forces, grey. [file 1471-2180-14-102-S6.docx]

*
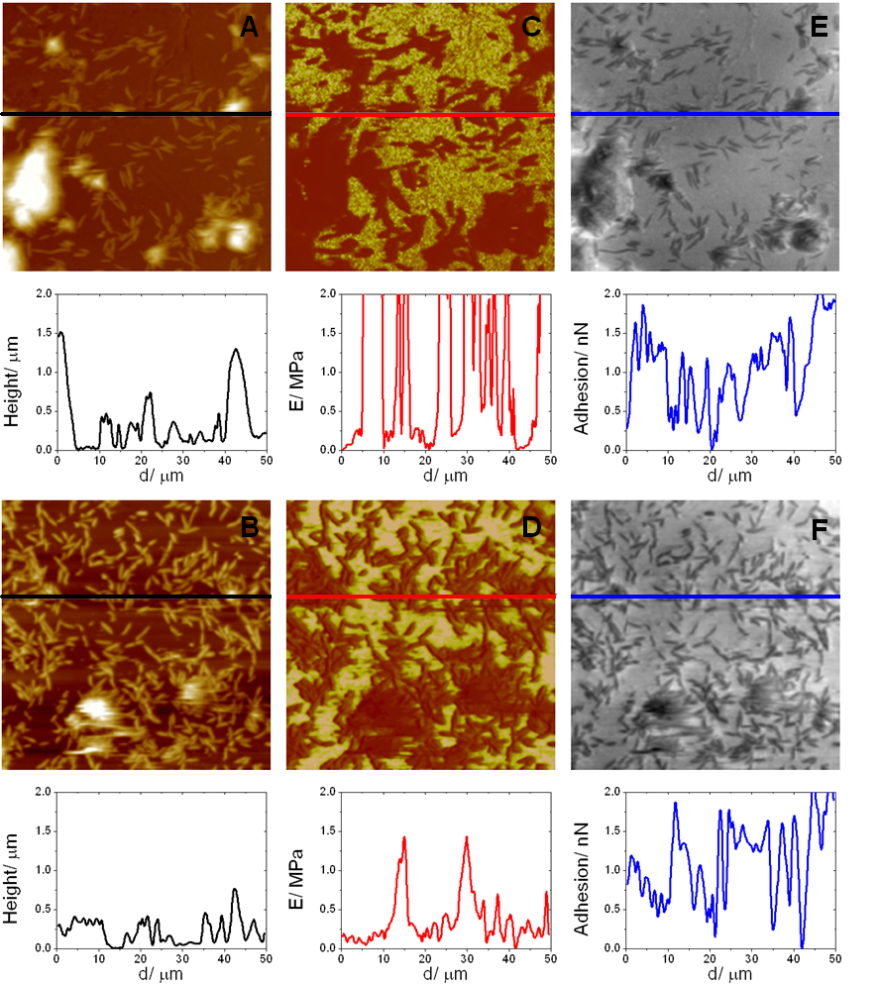
*

Supplement: Additional file 7: Figure S4 — Representative cross-sections of 2D Peak Force Tapping 50 x 50 μm2 images. (A) and (B), topographic images of media LMB and SASW, respectively, in brown; (C) and (D), Young’s modulus quantitative mappings, in gold; (E) and (F), adhesion forces, grey. [file 1471-2180-14-102-S7.docx]

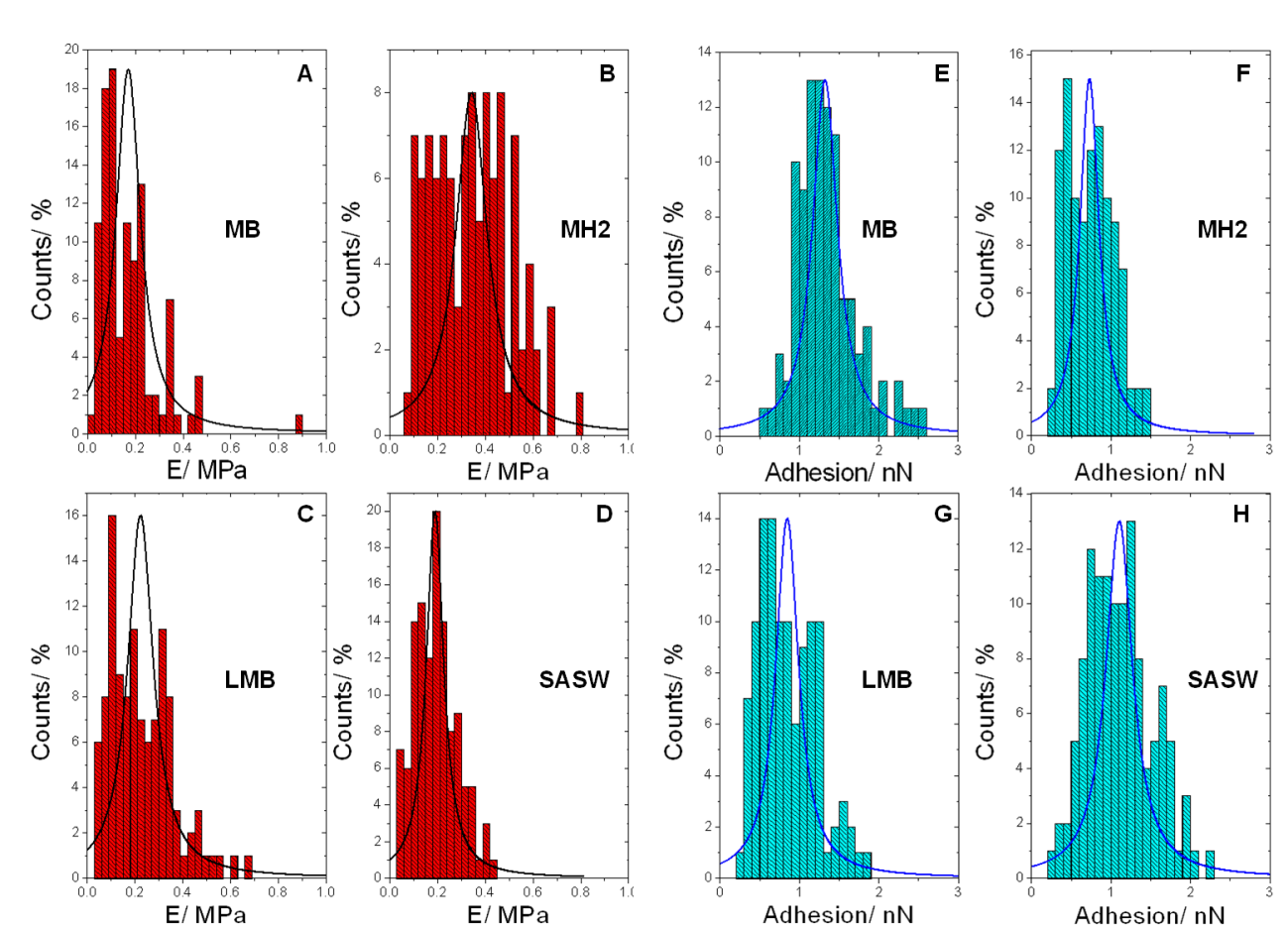

Supplement: Additional file 8: Figure S5 — Histograms showing the elastic modulus (E, red bars) and adhesion force (blue) distributions for Shewanella algae cells. (A) and (E) MB; (B) and (F) MH2; (C) and (G) LMB; (D) and (H) SASW. [file 1471-2180-14-102-S8.docx]

.


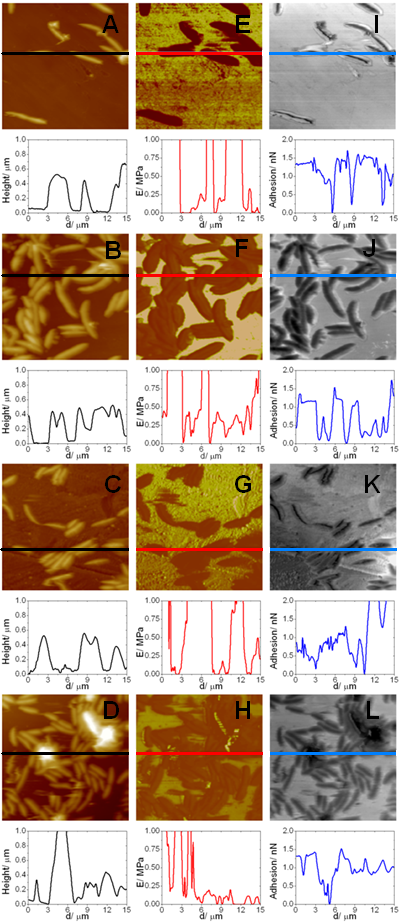

Supplement: Additional file 9: Figure S6 — Representative cross-section of 2D Peak Force Tapping 15 x 15 μm2 images. (A)-(B), topographic images of media MB, MH2, LMB, and SASW, respectively, in brown; (E)-(H), Young’s modulus quantitative mappings, in gold; (I)-(L), adhesion forces, grey. [file 1471-2180-14-102-S9.docx]

**
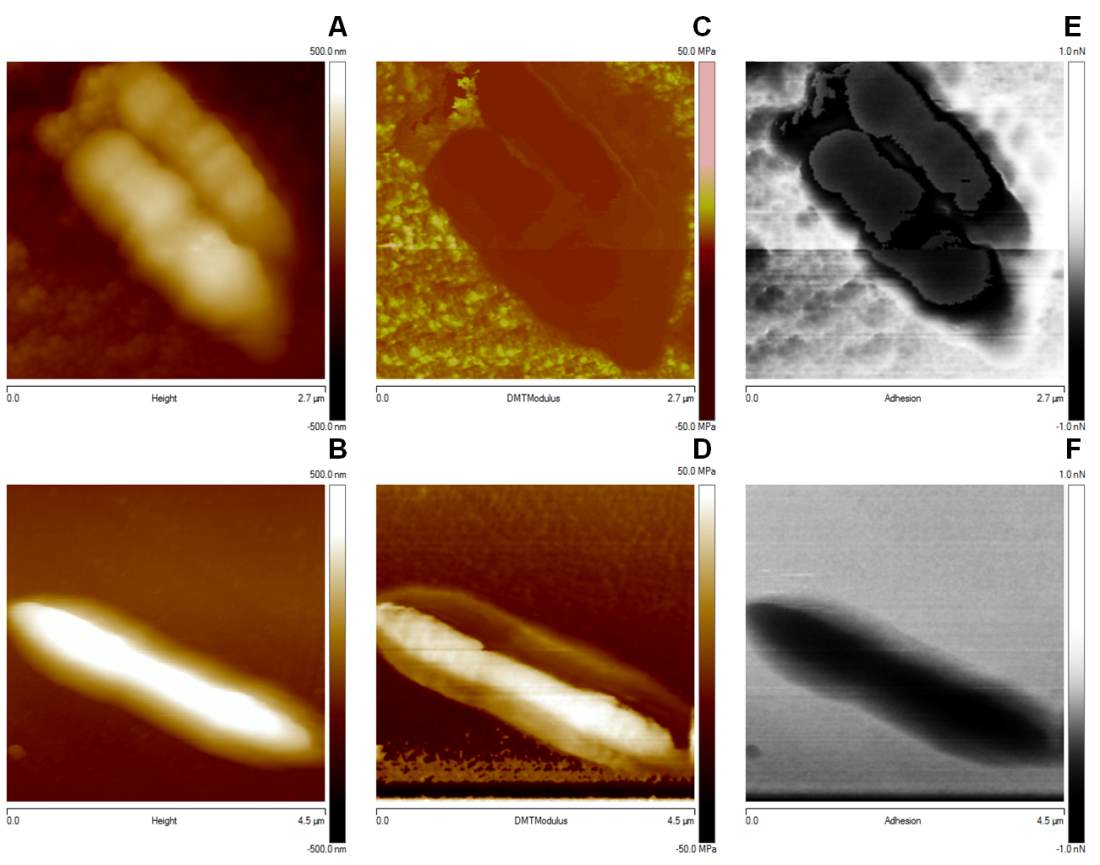
**

Supplement: Additional file 10: Figure S7 — Representative 2D Peak Force Tapping 2.7 x 2.7 μm2 (upper panel) and 4.5 x 4.5 μm2 (lower panel) images. (A) and (B), topographic images of media MB and MH2, respectively, in brown; (C) and (B), Young’s modulus quantitative, in gold; (E) and (F), adhesion forces, grey. [file 1471-2180-14-102-S10.docx]
