# Supplementary material for: On the influence of the culture conditions in bacterial antifouling bioassays and biofilm properties: Shewanella algae, a case study
Source: BMC Microbiol. 2014 Apr 23;14:102. doi: 10.1186/1471-2180-14-102 (PMC4021068; doi:10.1186/1471-2180-14-102)
Supplement: Additional file 4: Table S3 — One-way ANOVA and Welch ANOVA results for CLSM and AFM data, respectively. For the one-way ANOVA, the dependent variable has been logarithmic transformed to ensure homocedasticity. [file 1471-2180-14-102-S4.docx]

| **Variable** | **Significance** | **Multiple comparisons *p*-values** | |
| --- | --- | --- | --- |
| Mean Thickness | 0.000 | MB vs MH2 | 0.066 |
|  |  | MB vs LMB | 0.008 |
|  |  | MB vs SASW | 0.259 |
|  |  | MH2 vs LMB | 0.000 |
|  |  | MH2 vs SASW | 0.002 |
|  |  | LMB vs SASW | 0.214 |
| Max. Thickness | 0.000 | MB vs MH2 | 0.000 |
|  |  | MB vs LMB | 0.046 |
|  |  | MB vs SASW | 0.109 |
|  |  | MH2 vs LMB | 0.005 |
|  |  | MH2 vs SASW | 0.000 |
|  |  | LMB vs SASW | 0.001 |
| Coverage | 0.000 | MB vs MH2 | 0.206 |
|  |  | MB vs LMB | 0.000 |
|  |  | MB vs SASW | 0.024 |
|  |  | MH2 vs LMB | 0.003 |
|  |  | MH2 vs SASW | 0.595 |
|  |  | LMB vs SASW | 0.023 |
| Roughness coefficient | 0.000 | MB vs MH2 | 0.000 |
|  |  | MB vs LMB | 0.000 |
|  |  | MB vs SASW | 0.005 |
|  |  | MH2 vs LMB | 0.098 |
|  |  | MH2 vs SASW | 0.018 |
|  |  | LMB vs SASW | 0.000 |
| Young Modulus | 0.000 | MB vs MH2 | 0.000 |
|  |  | MB vs LMB | 0.001 |
|  |  | MB vs SASW | 0.165 |
|  |  | MH2 vs LMB | 0.000 |
|  |  | MH2 vs SASW | 0.000 |
|  |  | LMB vs SASW | 0.120 |
| Adhesion | 0.000 | MB vs MH2 | 0.000 |
|  |  | MB vs LMB | 0.000 |
|  |  | MB vs SASW | 0.000 |
|  |  | MH2 vs LMB | 0.045 |
|  |  | MH2 vs SASW | 0.000 |
|  |  | LMB vs SASW | 0.000 |
